# *In silico *analysis of methyltransferase domains involved in biosynthesis of secondary metabolites

**DOI:** 10.1186/1471-2105-9-454

**Published:** 2008-10-25

**Authors:** Mohd Zeeshan Ansari, Jyoti Sharma, Rajesh S Gokhale, Debasisa Mohanty

**Affiliations:** 1 National Institute of Immunology, Aruna Asaf Ali Marg, New Delhi-110067, India

## Abstract

**Background:**

Secondary metabolites biosynthesized by polyketide synthase (PKS) and nonribosomal peptide synthetase (NRPS) family of enzymes constitute several classes of therapeutically important natural products like erythromycin, rapamycin, cyclosporine etc. In view of their relevance for natural product based drug discovery, identification of novel secondary metabolite natural products by genome mining has been an area of active research. A number of different tailoring enzymes catalyze a variety of chemical modifications to the polyketide or nonribosomal peptide backbone of these secondary metabolites to enhance their structural diversity. Therefore, development of powerful bioinformatics methods for identification of these tailoring enzymes and assignment of their substrate specificity is crucial for deciphering novel secondary metabolites by genome mining.

**Results:**

In this work, we have carried out a comprehensive bioinformatics analysis of methyltransferase (MT) domains present in multi functional type I PKS and NRPS proteins encoded by PKS/NRPS gene clusters having known secondary metabolite products. Based on the results of this analysis, we have developed a novel knowledge based computational approach for detecting MT domains present in PKS and NRPS megasynthases, delineating their correct boundaries and classifying them as N-MT, C-MT and O-MT using profile HMMs. Analysis of proteins in nr database of NCBI using these class specific profiles has revealed several interesting examples, namely, C-MT domains in NRPS modules, N-MT domains with significant homology to C-MT proteins, and presence of NRPS/PKS MTs in association with other catalytic domains. Our analysis of the chemical structures of the secondary metabolites and their site of methylation suggested that a possible evolutionary basis for the presence of a novel class of N-MT domains with significant homology to C-MT proteins could be the close resemblance of the chemical structures of the acceptor substrates, as in the case of pyochelin and yersiniabactin. These two classes of MTs recognize similar acceptor substrates, but transfer methyl groups to N and C positions on these substrates.

**Conclusion:**

We have developed a novel knowledge based computational approach for identifying MT domains present in type I PKS and NRPS multifunctional enzymes and predicting their site of methylation. Analysis of nr database using this approach has revealed presence of several novel MT domains. Our analysis has also given interesting insight into the evolutionary basis of the novel substrate specificities of these MT proteins.

## Background

Nonribosomal peptide synthetases (NRPSs), polyketide synthases (PKSs) and fatty acid synthases (FASs) employ a common biosynthetic strategy to synthesize their metabolic products by stepwise condensation of simple amino or carboxylic acid monomers. The core catalytic domains involved in the biosynthesis of the polyketide/nonribosomal peptide/fatty acid backbone moieties are ketosynthase (KS), acyltransferase (AT), dehydratase (DH), enoylreductase (ER), ketoreductase (KR), acyl carrier protein (ACP), condensation (C), adenylation (A) and thiolation (T) [1,2]. Apart from these core catalytic domains, a number of auxiliary functional domains, often called tailoring domains, introduce a variety of different chemical modifications to the backbone moieties of these secondary metabolites to further increase their structural diversity. Bioinformatics analysis of various catalytic domains present in NRPS and PKS proteins has been an area of active research in recent years [3-8]. These studies [3-8] have not only led to development of novel computational methods for *in silico *identification of secondary metabolites by genome mining [9-16], they have also guided rational reprogramming of secondary metabolite biosynthetic pathways to generate designed "natural products" [12,17-20]. However, all these studies including our earlier work have concentrated on core catalytic domains and no detailed bioinformatics analyses have been carried out for important tailoring enzymes like, methyltransferases.

Methyltransferase (MT) domains present in NRPS and PKS clusters constitute a major class of tailoring domains/enzymes involved in biosynthesis of secondary metabolites. They catalyze the transfer of methyl group from S-adenosylmethionine (SAM or AdoMet) to the carbon, nitrogen or oxygen atoms at various positions on the backbones of polyketides, nonribosomal peptides and fatty acids and therefore have been classified as C-MT, N-MT and O-MT respectively depending upon their site of methylation. These enzymatic domains in general have a bidomain structure, where the first subdomain contains the binding site for methyl group donor, while the second subdomain harbors the binding site for acceptor substrate [21,22]. The presence of MT domains in multifunctional NRPS and PKS proteins is generally inferred from chemical structure of the secondary metabolite products. There are only few in vitro studies on enzymatic characterization of NRPS/PKS MT domains [23-27]. A recent study on MT domains from type II PKS biosynthetic pathways has revealed interesting correlation between regioselectivity of methylation and MT sequence [24]. However, no such analysis has been carried out for MT domains present in type I PKS or NRPS proteins. In contrast to type II PKS MTs which are stand alone proteins, MT domains in type I PKS and NRPS are present along with other catalytic domains on a single polypeptide chain. Therefore, it has been difficult to decipher the correct length and domain boundaries for MT domains in type I PKS or NRPS proteins. Various studies have suggested that the size of N-MT domain is typically 450 amino acids, while C-MT and O-MT are generally 300 amino acids long. A set of 3 conserved sequence motifs has been identified in most MTs [28-30]. Mutational studies of N-MTs of peptide synthetases have shown that these 3 motifs are essential for the catalysis [31]. The knowledge of these MT sequence motifs and the expected spacing between them is often used for discerning presence of MT domains in multifunctional NRPS and PKS proteins. However, because of the high degree of sequence divergence, delineating the correct boundary of these proteins is quite often a difficult task. In our earlier study, we attempted to identify MT domains in various NRPS/PKS gene clusters based on pairwise alignment with MT domain from actinomycin cluster [32]. However, this domain identification protocol failed to detect 23 out of 32 MT domains. The 23 unidentified MT domains included the three groups of MTs (C-, O- and N-MTs), for which proper templates were not available. The general purpose domain identification tools like CDD-search can identify MT domains in NRPS and PKS proteins, but can not predict the domain boundaries accurately and they also fail to classify them as C-MT, N-MT and O-MT. Such classification is crucial for prediction of chemical structures of secondary metabolites. The knowledge of substrate specificity and domain boundaries of MT domains is also important for rational design of novel secondary metabolites by introduction of heterologous MT domains.

In this manuscript, we have carried out a systematic analysis of the sequence/structural features of MT domains present in various experimentally characterized NRPS and type I PKS clusters having known metabolic products. Since crystal structures are available for many stand alone small molecule methyltransferases from several microbial organisms, we have carried out threading analysis for the experimentally characterized MT domains from NRPS and PKS biosynthetic pathways. The threading analysis has helped in elucidating the putative three dimensional structure adopted by MT domains and based on the alignment of MT containing sequences on the structural fold of MT domain it has been possible to delineate the correct boundaries for NRPS/PKS MT domains. Our threading analysis has also given novel insight into the structural features of linker sequences flanking the MT domains in NRPS and PKS proteins. Using the curated sequences of these MTs, we have carried out detailed phylogenetic analysis to investigate whether these catalytic domains cluster as per their specificity for site of methylation i.e. C-MT, N-MT and O-MT. Based on this analysis, we have identified suitable template sequences of C-MT, N-MT and O-MT domains from representative clusters, which can be used to identify MT domains in uncharacterized NRPS/PKS proteins. We have also developed Hidden Markov Model (HMM) profiles which can identify MT domains in a query sequence and classify them as N-MT, C-MT and O-MT. Using these HMM profiles, we have analyzed non-redundant protein sequence database of NCBI to identify other multifunctional enzymes containing C-MT, N-MT and O-MT domains.

## Results

**Figure 1:**
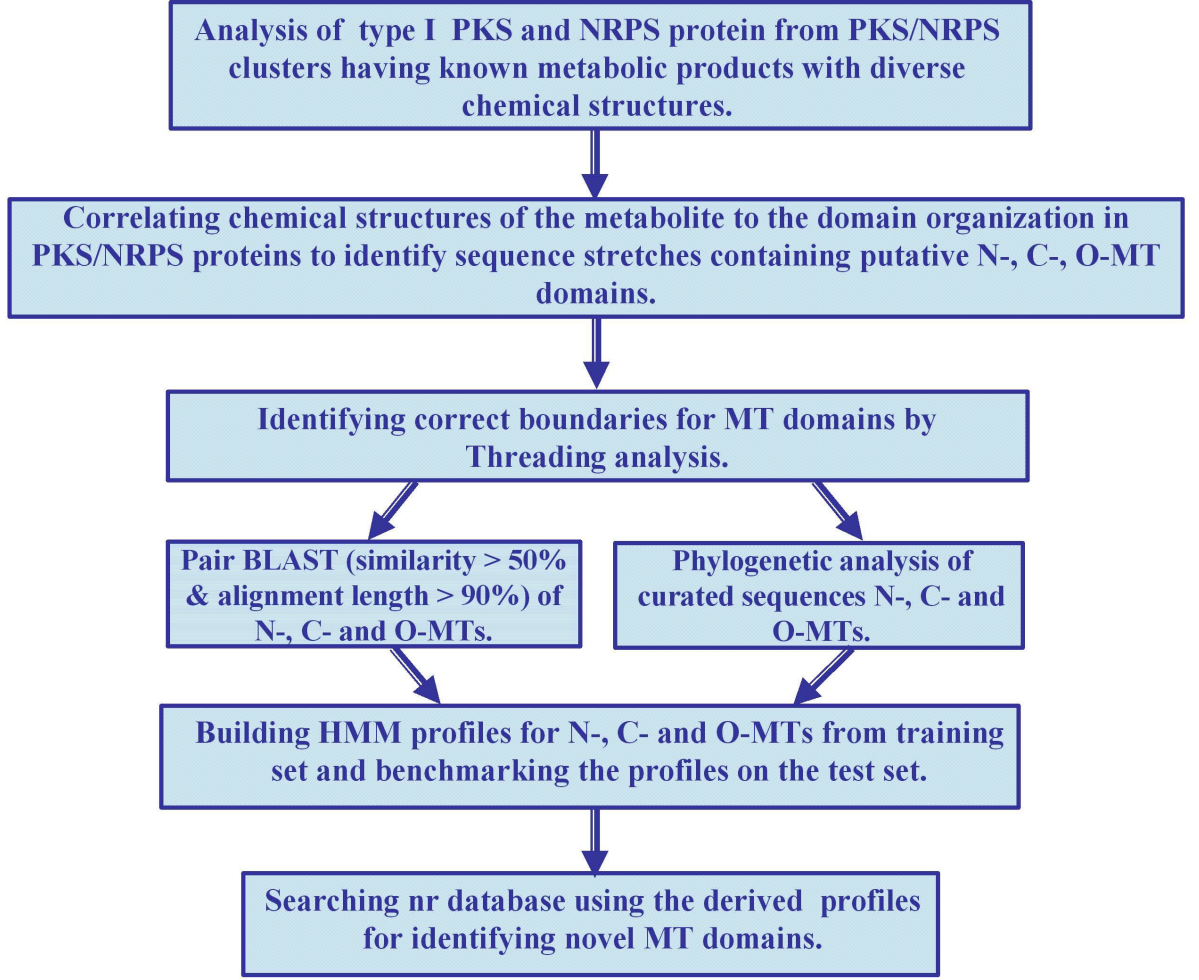
A schematic overview of different bioinformatics analyses carried out in the current work on MT domains present in type I PKS and NRPS proteins.

In this study, we have carried out a number of different bioinformatics analyses on MT domains present in type I PKS and NRPS proteins, to correlate the sequence of these MT domains to their substrate specificity i.e. the site of methylation. Figure 1 gives a schematic overview of the various different analyses carried out and the type of results obtained from them, while the results are discussed in detail in the following sections.

**Figure 2:**
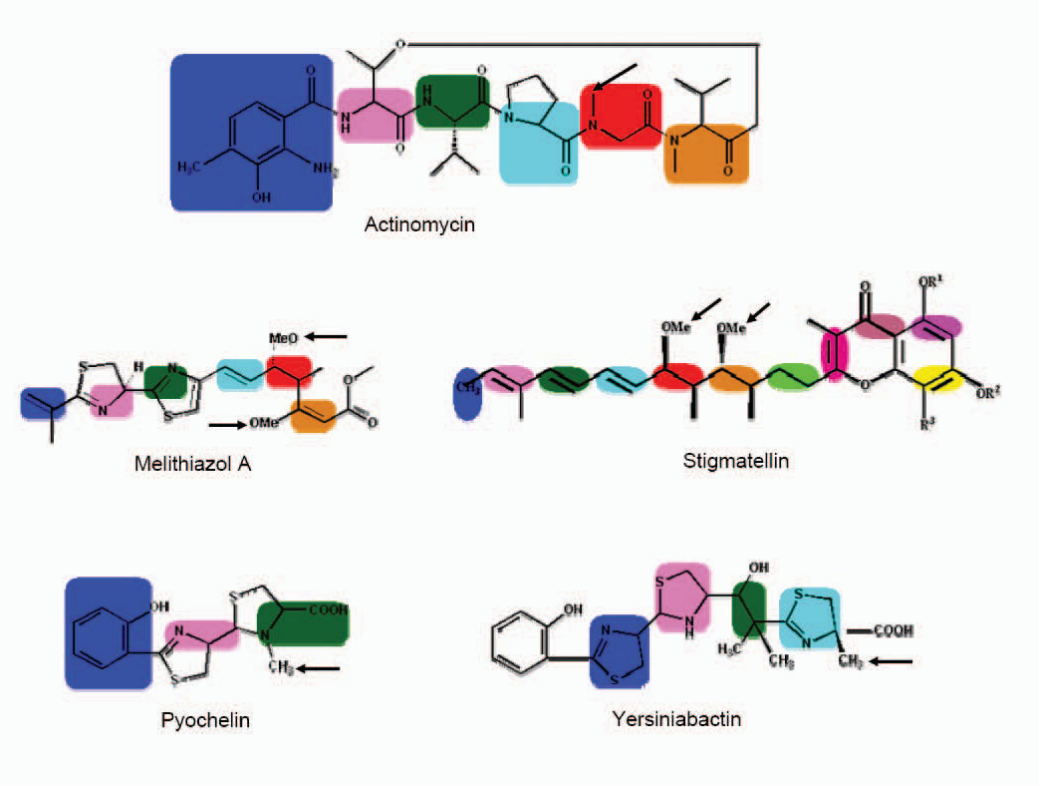
Chemical structures of representative secondary metabolites like nodularin, leinamycin, pyochelin, yersiniabactin and stigmatellin containing methyl groups (highlighted by arrow sign) added by C-MT, N-MT and O-MT enzymatic domains.

**Figure 3:**
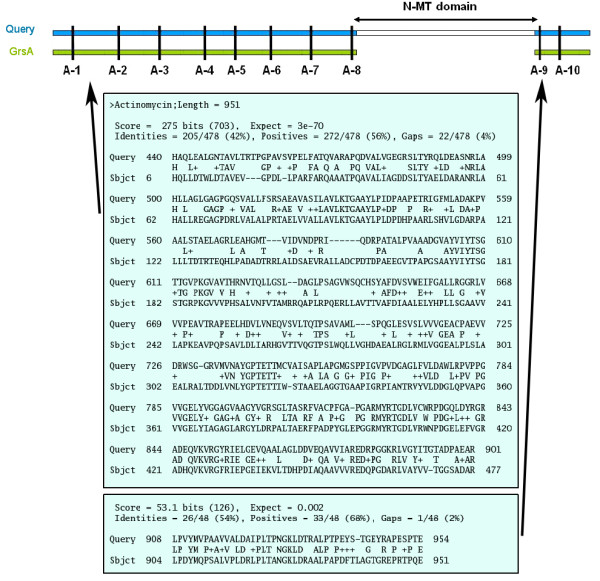
**Alignment of the sequence stretch containing A and N-MT domains from a C-A-MT-T NRPS module with sequence of A domain from a C-A-T module.** A split alignment is obtained, because N-MT domain is integrated between A-8 and A-9 signature motifs of A domain.

The chemical structures of the secondary metabolites produced by various PKS, NRPS and hybrid NRPS/PKS clusters cataloged in NRPS-PKS web resource were analyzed carefully to identify methyl substitutions on polyketide or nonribosomal peptide backbones. The presence of methyl substitutions on nitrogen and oxygen atoms indicated presence of N-MT or O-MT domains in the proteins encoded by these gene clusters. However, in absence of MT domains methyl substitutions on carbons in a ketide group can also result from selection of methylmalonate extender groups by the AT domains of PKS proteins. Therefore, for correctly inferring presence of C-MT domains in a PKS protein, the substrate specificity of the corresponding AT domain was also checked. Table 1 lists various ORFs harboring the MT domains, their GenBank accession number and the type or substrate specificity of MT domain as deduced from the chemical structure of the metabolite. As can be seen, the data set consisted of 20 C-MT, 19 O-MT and 22 N-MT domains from 27 different NRPS/PKS clusters. Figure 2 shows the chemical structures of 5 representative secondary metabolites highlighting the methyl groups added by C-MT, N-MT and O-MT domains, while chemical structures of the remaining 22 secondary metabolites are shown in Additional file 1. Each of the ORFs listed in Table 1 were analyzed by NRPS-PKS search tool as well as CDD server of NCBI. NRPS-PKS search tool, which used a single MT domain from actinomycin cluster as template, could identify only 26 MT domains out of a total of 61. Even though the latest version of CDD server could identify 55 out of these 61 MT domains, the lengths of the MT domains detected by both these programs were notably shorter than the typical length of these domains. These programs also failed to distinguish between C-MT, N-MT and O-MT domains. Additional file 2 shows the length of each MT containing sequence stretch and other catalytic domains flanking this region. As can be seen, all N-MT domains are present in NRPS clusters only as C-A-MT-T modules and typically a 400 amino acid sequence stretch containing this domain is inserted in the adenylation domain between the conserved motifs A8 and A9 of adenylation domains and hence alignment of these N-MT containing A domains with regular A domains produces a split alignment (Figure 3). All stand alone O-MT containing sequences were typically 300 to 400 amino acids long, while the amino acid stretches containing other O-MT and C-MT domains present in type I PKS proteins were 600 to 700 amino acids long. The O-MT and C-MT domains in PKS proteins were present in four different types of modules i.e. KS-AT-MT-ACP, KS-AT-MT-KR-ACP, KS-AT-DH-MT-KR-ACP and KS-AT-DH-MT-ER-KR-ACP. Only in case of leinamycin gene cluster, which has trans-AT domains, the MT domain is present as KS-DH-KR-ACP-MT-ACP. There were only two examples where MT domain was adjacent to an aminotransferase (AMT) domain in a hybrid NRPS/PKS system. In these cases MT-AMT stretch was inserted between a PKS and a NRPS module. It appears that MT domains in type I PKS proteins are present in AT-KR, DH-ER or DH-KR linker regions which are typically more than 200 amino acids long. Hence, length of the flanking linker region could be the reason for the larger length of these MT containing sequence stretches. Therefore, we decided to carry out various structure based sequences analysis for representative MT containing stretches of each category to delineate the exact domain boundaries for MT domains.

**Table 1:** List of ORFs containing C-, O- and N-Methyltransferase domains

Name of gene cluster	ORF	Accession no.	CDD search	Types of MT-domain			Total
				C-MT	O-MT	N-MT	

				NRPS clusters			

Actinomycin	acmC	AAF42473	PF08242	-	-	2	2
Anabaenopeptilide	apdB	CAC01604	PF08242	-	-	2	
	apdE	CAC01607	PF08241	-	1	-	3
Complestatin	comC	AAK81826	PF08242	-	-	1	1
Cyclosporine	simA	CAA82227	PF08242	-	-	7	7
Enniatin	esyn1	CAA79245	PF08242	-	-	1	1
Pristinamycin	snbDE	T30289	PF08242	-	-	1	1
Pyochelin	pchF	AAD55801	PF08242	-	-	1	1
Thaxtomin	txtA	AAG27087	PF08242	-	-	1	
	txtB	AAG27088	COG2226	-	-	1	2

				PKS clusters			

Compactin	mlcA	BAC20564	PF08242	1	-	-	
	mlcB	BAC20566	PF08242	1	-	-	2
Erythromycin	eryG	CAA42929	COG2226	-	1	-	1
Equisetin	eqiS	AAV66106	PF08242	1	-	-	1
Fumonisin	fum1	AAD43562	PF08242	1	-	-	1
Lovastatin	lovB	Q9Y8A5	PF08242	1	-	-	
	lovF	AAD34559	PF08242	1	-	-	2
Stigmatellin	stiD	CAD19088	PF08242	-	1	-	
	stiE	CAD19089	PF08242	-	1	-	
	stiK	CAD19094	PF01209	-	1	-	3

				Hybrid NRPS-PKS			

Bleomycin	blmVIII	AAG02357	PF08242	1	-	-	1
Barbamide	barF	AAN32980	PF08242	-	1	-	
	barG	AAN32981	PF08242	-	-	1	2
Epothilone	epoD	AAF26922	PF08242	1	-	-	1
Jamaicamide A	jamJ	AAS98781	PF08242	1	-	-	
	jamN	AAS98785	COG0500	-	1	-	2
Leinamycin	lnmJ	AAN85523	PF08242	1	-	-	1
Melithiazol	melE	CAD89776	PF08242	-	1	-	
	melF	CAD89777	PF08242	-	1	-	2
Microcystin	mcyD	BAB12210	PF08242	1	-	-	
	mcyE	BAB12211	-	1	-	-	
	mcyG	BAB12213	PF08242	1	-	-	
	mcyA	BAA83992	PF08242	-	-	1	4
Myxothiazol	mtaE	AAF19813	PF08242	-	1	-	
	mtaF	AAF19814	PF08242	-	1	-	2
Nodularin	ndaC	AAO64404	-	1	-	-	
	ndaD	AAO64405	PF08242	1	-	-	
	ndaF	AAO64407	-	1	-	-	
	ndaA	AAO64403	PF08242	-	-	1	
	ndaE	AAO64406	PF08242	-	1	-	5
Onnamide	onnB	AAV97870	PF08242	-	1	-	
	onnD	AAV97872	PF08242	-	1	-	
	onnG	AAV97875	PF08242	-	1	-	
	onnH	AAV97876	COG2226	-	1	-	
	onnI	AAV97877	PF08242	-	1	-	5
Pederin	pedF	AAS47564	PF08242	1	-	-	
	pedA	AAS47557	PF08241	-	1	-	
	pedE	AAS47560	COG2226	-	1	-	3
Tubulysin	tubF	CAF05651	PF08242	1	-	-	
	tubB	CAF05647	PF08242	-	-	1	
	tubC	CAF05648	PF08242	-	-	1	3
Yersiniabactin	HMWP-1	AAC69588	PF08242	2	-	-	2

Total				20	19	22	61

List of ORFs containing methyltransferase domains in various experimentally characterized NRPS/PKS gene clusters. Table also lists the number and type of MT domains in each of the ORFs and results from PFAM analysis using CDD search.

### Threading analysis of MT domains

Since domain boundaries can be identified correctly by aligning the sequence of multi domain proteins with the 3D structures of the corresponding single domain proteins, we attempted to identify other proteins in PDB which are structurally similar to these MT domains of PKS/NRPS enzymes. However, the lack of crystal structures for any MT domains from PKS/NRPS biosynthetic pathway and high degree of sequence divergence in this enzyme family prompted us to use threading or fold recognition approach. These tools can potentially reveal structural similarity in absence of high degree of similarity in sequences. GenTHREADER and PHYRE fold prediction servers were used for threading analysis. As discussed in the methods section, the MT sequence stretch identified by CDD or NRPS-PKS along with their flanking linkers was threaded on various structural folds in PDB. In cases where chemical structure of metabolite indicated presence of MT domains but no MT domain was detected by these programs, all linker stretches having unusual length were analyzed by both these fold recognition servers.

Table 2 shows the results of threading analysis for representative members of these MT containing sequences. The fold prediction hits with highest level of statistical significance corresponding to p-value lower than 0.0001 were labeled as CERTAIN by the GenTHREADER server, while hits having p-value between 0.0001 and 0.001 are labeled as HIGH. We considered only those matches which are labeled as CERTAIN or HIGH by GenTHREADER or have precision of more than 95% in case of fold prediction by PHYRE. As can be seen, all the 18 sequences matched with structure of MT proteins in PDB. This suggests that, the MT domains present in NRPS/PKS proteins would adopt a fold similar to the small molecule methyltransferase in other organisms. The N-MT domains present in our data set aligned not only with N-MT structures, but also with C-MT and O-MT structures. Similar was the case for C-MT and O-MT domains in our data set. Analysis of their alignment scores did not show any preference for structural matches from the same functional category. Therefore, the structures which aligned consistently with all the sequences by both servers and had maximum sequence identity and alignment length with the query sequences were chosen as structural templates for MT domains of NRPS/PKS proteins. The structure of histamine N-MT (PDB code 1VLM; 207 residues) and a hypothetical protein from M. tuberculosis (PDB code 1VL5; 230 residues) showed alignment with most C-MT, O-MT and N-MT by both the fold prediction servers. However, taking into consideration the alignment length and the length of the query sequence, for further analysis 1VLM was selected as template for C-MT and O-MT domains, while 1VL5 was selected as structural template for N-MT domains. These results suggest that, MT domains present in type I PKS and NRPS proteins are likely to be around 200 amino acids long.

**Table 2:** Threading analysis of 18 representative MT containing sequences

**Methyltransferases**	**Len.**	**2hg4**	**1qzz**	**1vl5**	**1vlm**	**1wzn**	**2gpy**	**2aot**	**1g6q**	**1xxl**	**1im8**	**2fr0**
		**917**	**374**	**260**	**219**	**252**	**233**	**292**	**328**	**239**	**244**	**486**

melit01_OM_001	609	C	-	C {100}	C	-	C	-	-	-	-	C
anaba01_OM_001	263	-	-	C {100}	C {100}	C	-	C	-	{100}	-	-
onnam04_OM_001	268	-	-	C {100}	C	C	-	-	-	{100}	-	-
peder01_OM_001	312	-	-	C {100}	C	-	-	-	-	{100}	-	-
stigm03_OM_001	256	-	-	C {100}	C {100}	C	-	-	-	{100}	-	-
eryth01_OM_001	306	-	-	C {100}	C	-	C	-	-	{100}	-	-
barba01_OM_001	422	-	-	C {100}	C	C	C	-	-	{100}	-	-
onnam01_OM_001	484	-	-	{100}	C {100}	-	-	C	-	{100}	{100}	-
bleom01_CM_001	640	C	-	C {100}	C {100}	C	C	-	-	{100}	{100}	C
nodul02_CM_001	720	-	C	C {100}	C {100}	-	C	C	-	{100}	-	C
compa01_CM_001	490	-	-	C {100}	C {100}	-	C	C	-	{100}	-	-
leina01_CM_001	432	-	C	C {100}	C {100}	C {100}	C	C	-	-	-	-
yersi01_CM_002	479	-	C	C	C	-	C	C	-	-	{95}	-
actin01_NM_001	422	-	-	{100}	{100}	C {100}	C	-	-	{100}	-	-
anaba01_NM_001	367	-	-	-	-	-	C	-	H {100}	-	-	-
cyclo01_NM_001	431	-	-	{100}	{100}	-	H	-	-	{100}	-	-
pyoch01_NM_001	390	-	H	{100}	C {100}	-	C	C	-	{100}	{100}	-
thaxt02_NM_001	362	-	-	{100}	C {100}	C	C	-	-	{100}	-	-

Column 1 gives the unique name assigned to each MT containing sequence stretch, while the second column indicates their length. Column 3–13 lists the PDB IDs for the structures which show alignment with these sequences in fold recognition analysis using GenTHREADER and PHYRE servers. Results from GenTHREADER corresponding to confidence level CERTAIN and HIGH are labeled as C and H respectively. Similarly, PHYRE results corresponding to precision level 100% and 95% are labeled as 100 and 95 respectively in curly braces. (-) indicates the absence of that particular fold.

**Figure 4:**
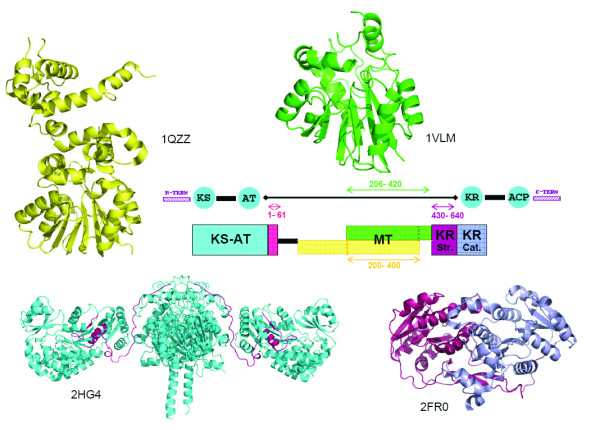
**Schematic representation of the results of threading analysis for typical C-MT containing sequence (from bleomycin ORF blmVIII) stretches having large length.** The central stretch aligns with various methyltransferase crystal structures like 1VLM and 1QZZ. A 200 amino acid C-terminal stretch aligns with the structural half of the KR domain in the crystal structure 2FR0, a 60 amino acid N-terminal stretch shows alignment with the terminal stretch of the KS-AT di-domain structure 2HG4. The query sequence containing the MT domain is represented as a black line, while rectangular colored boxes represent matches with various structural folds. The corresponding structures are shown in the same color.

**Figure 5:**
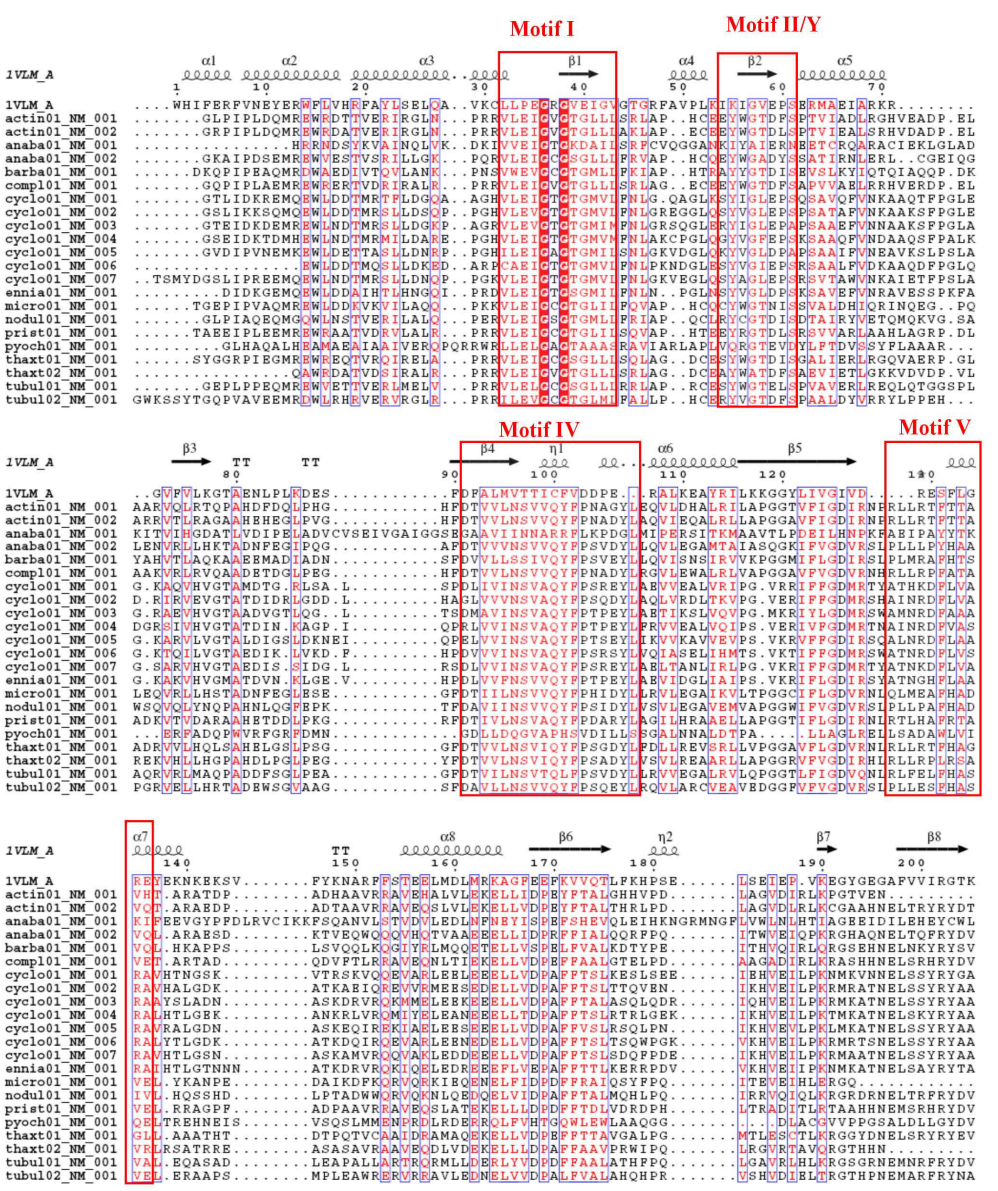
Multiple sequence alignments of N-MT domains from experimentally characterized NRPS/PKS clusters with the structural template 1VLM.

**Figure 6:**
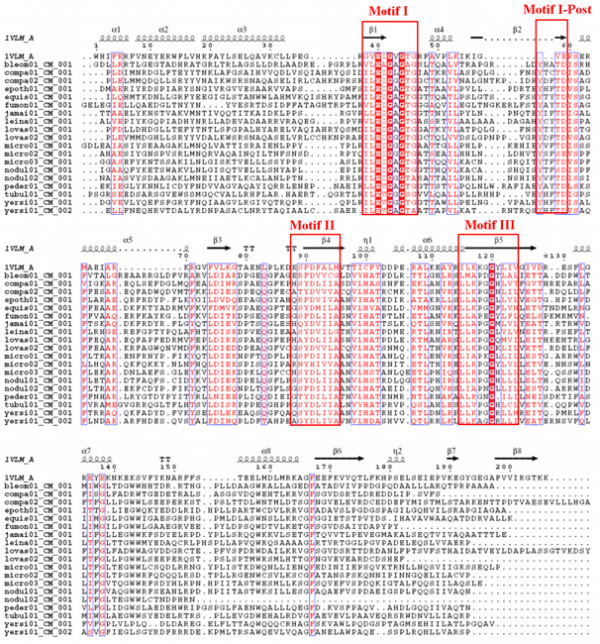
Multiple sequence alignments of C-MT and domains from experimentally characterized NRPS/PKS clusters with the structural template 1VLM.

**Figure 7:**
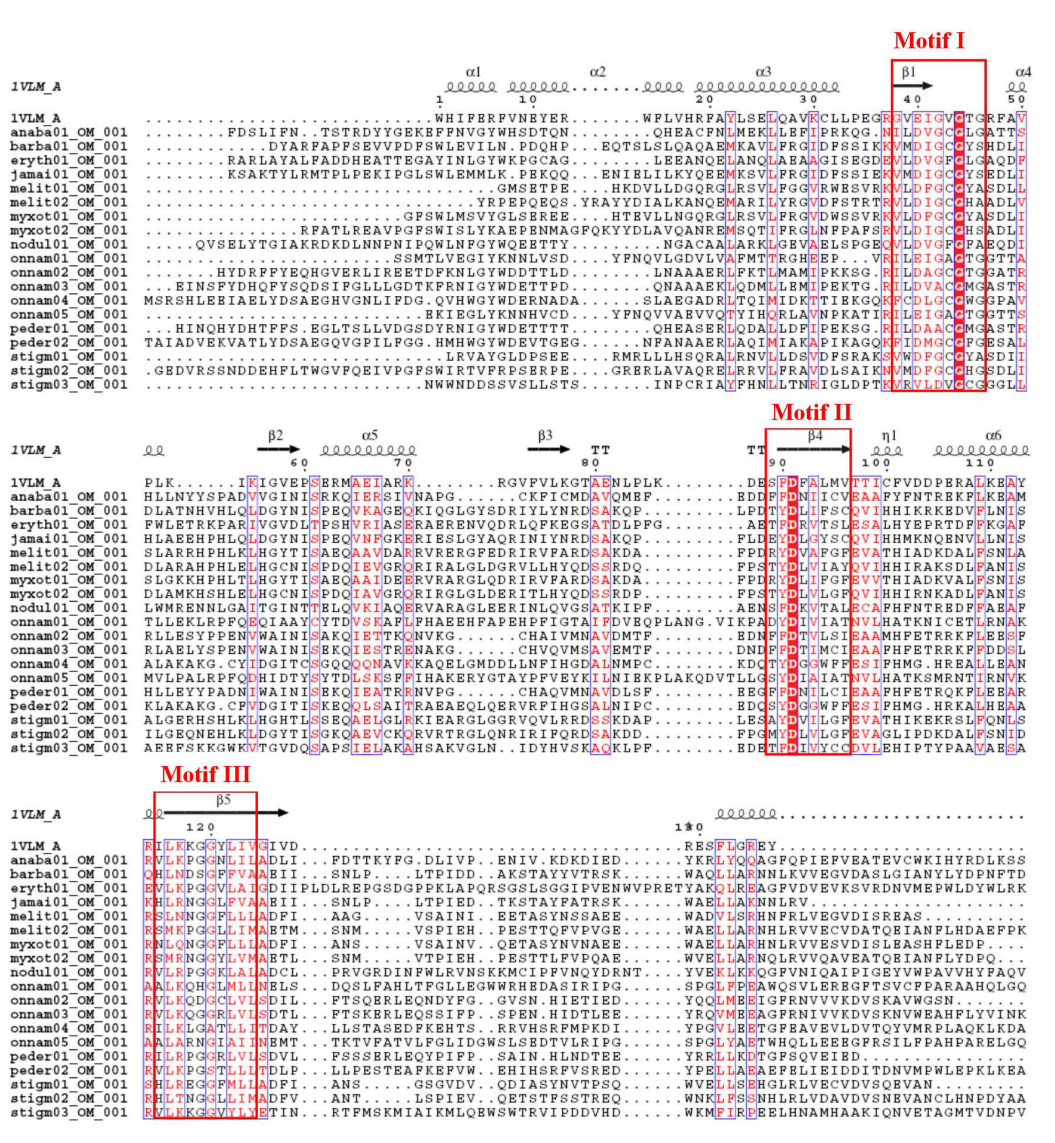
Multiple sequence alignments of O-MT domains from experimentally characterized NRPS/PKS clusters with the structural template 1VLM.

It may be noted that the small molecule MT structures present in PDB show significant variation in their length and in case of some of these sequences alignments were found with MT structures having significant difference in length. For example, 3 of the C-MT domains and 1 N-MT domain in our data set aligned with aclacinomycin-10-hydroxylase (PDB code 1QZZ; 340 residues) [33] and histamine N-MT (PDB code 1VLM; 207 residues). The huge length difference between these two MT structures made the choice of correct structural template a difficult task. 1QZZ aligns with the beginning of the MT containing sequence stretch, while in the alignment of the same sequences with 1VLM, the length of the linker preceding the MT domain ranges from 50 to even 200 residues (Figure 4). However, careful manual analysis of the alignments as well as the corresponding structural templates indicated that, the crystal structure 1QZZ consists of a predominantly α-helical N-terminal domain which is involved in dimerization and a C-terminal SAM binding domain typical of methyltransferases. On the other hand, the crystal structure 1VLM contains the SAM dependent MT domain alone. The overlapping region of both the alignments correspond to the catalytic domain of methyltransferase, while the linker sequences preceding the MT catalytic domain showed alignment with additional dimerization domains, if present in the structural template. Earlier studies on structural analysis of small molecule methyltransferases have suggested that, these enzymes have a conserved SAM-MT fold consisting of alternating β strands and α helices [22]. Earlier bioinformatics analysis [28,30,31] of MT domains has also revealed that, despite high divergence in primary sequence, certain conserved sequence motifs are present in all SAM dependent methyltransferases. Therefore, we wanted to examine whether the conserved secondary structural elements and sequence motifs responsible for catalytic activity, substrate specificity and cofactor binding are conserved in the MT domains identified by threading alignments. Figures 5, 6 and 7 show the structure guided multiple alignment of representative N-MT, C-MT and O-MT sequences with the structural template 1VLM. As can be seen from Figure 5, the N-MT sequences have the conserved motifs I, II/Y, IV and V in N-MTs. Similarly motifs I, motif I-post, II and III are present in the C-MT and O-MT sequences (Figures 6 &7). Thus all the motifs [28,30,31] identified in earlier analysis of small molecule methyltransferase sequences were found to be conserved in the multiple sequence alignments of C-, O- and N-MT domains identified by our threading analysis. The average percent identity among the C-, O- and N-MT domains was found to be 31, 28 and 17% respectively.

**Figure 8:**
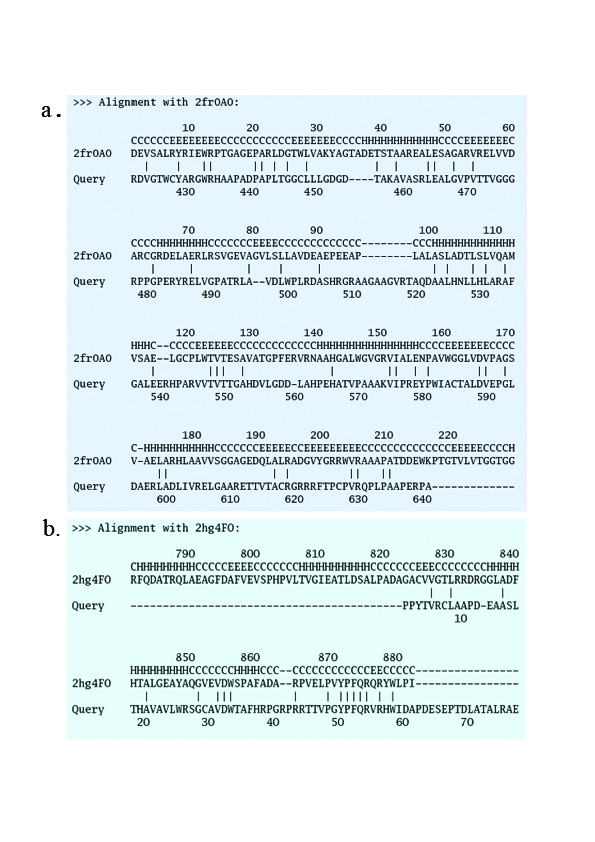
**Threading alignment C-MT containing sequence (ORF blmVIII) stretch from bleomycin gene cluster.** (a) Alignment of 60 amino acid N-terminal stretch with structure of KS-AT di-domain (2HG4) from erythromycin PKS (b) Alignment of 200 amino acid C-terminal region with structure (2FR0) of KR domain from erythromycin PKS.

The threading analysis also showed statistically significant matches with proteins other than methyltransferases. Such matches were specifically seen for MT containing sequences which were longer in length due to the presence of large flanking linker sequences. As can be seen from table 2 and figure 4, the N-terminal region of MT containing sequence from bleomycin showed an alignment (Figure 8a) with the last 60 amino acids of the KS-AT di-domain structure from erythromycin PKS [34]. This stretch corresponds to the last helix of the AT domain and a segment of the AT-DH linker region (Figure 4). The C-terminal 200 amino acid stretch of the MT containing sequence from bleomycin [35], nodularin and melithiazol also showed highly significant alignments (Figure 8b) with the structural subdomain (Figure 4) of the recently elucidated structure of KR from erythromycin PKS. These results suggest that, in type I PKS proteins, the linker sequences preceding the MT domain i.e. AT-MT linkers are homologous to the AT-DH linkers and MT-KR linker regions are likely to adopt a short chain reductase (SCR) fold and would constitute the structural half of the KR domain as demonstrated in erythromycin PKS. Thus MT catalytic domain is likely to be 200 amino acids only.

### Development of a computational protocol for identifying MT domains and their classification as C-MT, O-MT and N-MT

**Figure 9:**
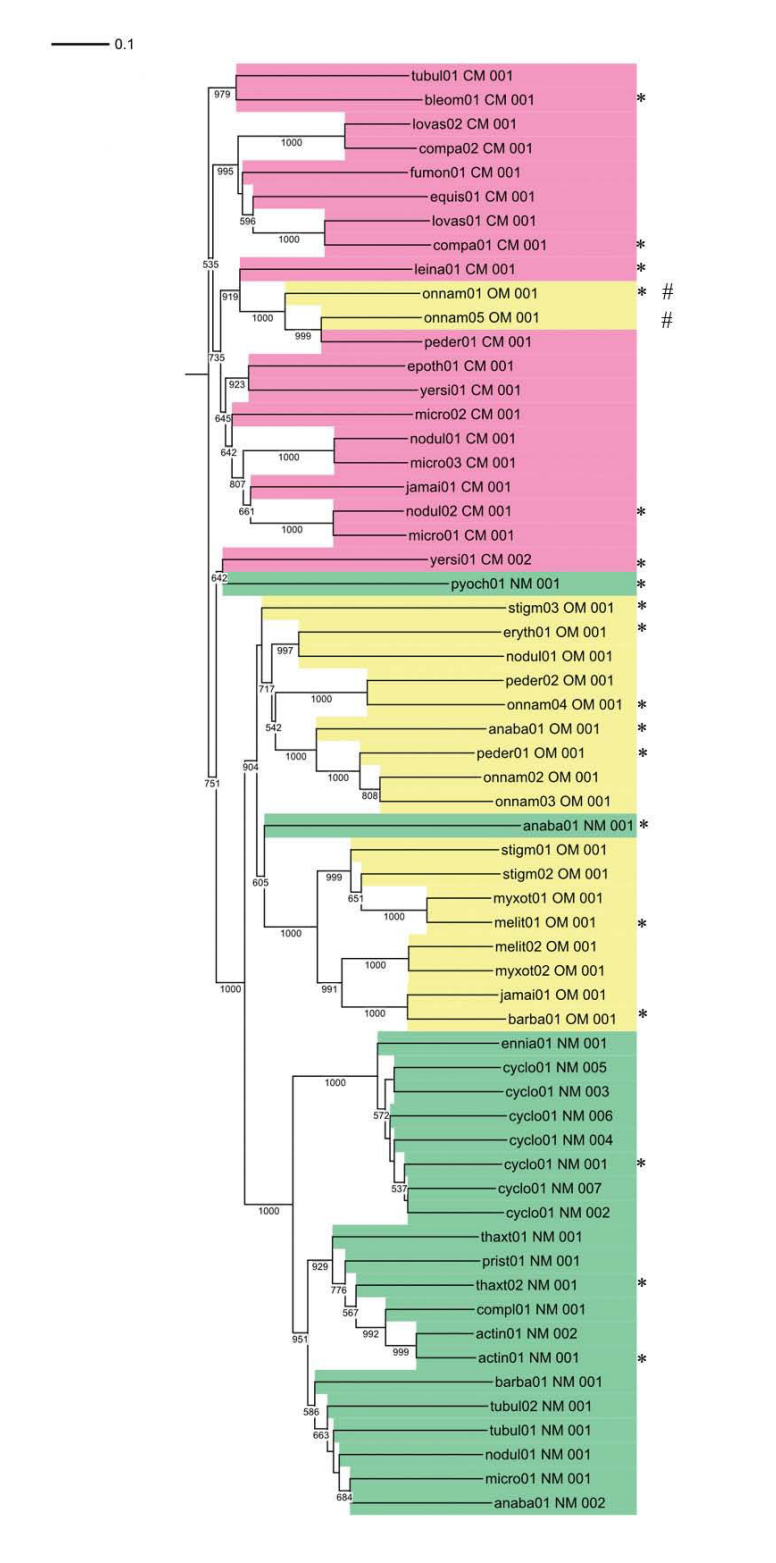
**Dendrogram of 60 MT domains from experimentally characterized NRPS, PKS and hybrid NRPS/PKS biosynthetic clusters.** The C-MT, O-MT and N-MT are colored pink, yellow and green respectively. The 18 representative MT sequences used as templates for detecting MT domains in a query are marked by "*". Two MT domains from onnamide-A which are annotated as O-MTs and cluster with C-MTs are marked with hash (#) symbol.

The domain boundaries were thus identified with 1VLM (for C- and O-MT) and 1VL5 (for N-MT) as templates and the regions which aligned with these templates were extracted from the 61 MT domain sequences. They represented the curated MT domains with correct boundary. A set of 42 MT domains out of these 60 sequences were used as test set to check if MT domains can be correctly classified by pairwise alignment with these 18 template sequences. Each of the 42 MT domains was queried against 18 MT templates to find the number of MT domains identified by these templates. The query MT domain was classified as C-MT, O-MT or N-MT depending on the highest scoring match from the template set. The results obtained by the pairwise comparison of 60 query sequences with the 18 template sequences indicated that C-MT templates were able to identify all other C-MT sequences. However, there were a few O- and N-MTs, which were also recognized by these C-MT templates. Specifically, 2 MT sequences in onnamide gene cluster (onnB and onnI) showed very high similarity to C-MT while they are functionally annotated as O-MTs by the authors who reported experimental characterization of this gene cluster [36]. These 2 O-MTs also have motif I as ExGxG which is characteristic of C-MTs. An N-MT from pyochelin synthetase (pchF) also exhibited considerable similarity to several C-MTs and the two onnamide O-MTs. In view of this apparent anomaly in sequence similarity of these proteins, their functional assignment needs to be examined carefully. In the ORF apdB of anabaenopeptilide gene cluster, there are two MT-domains wherein the first one is entirely different from all the other MT sequences and does not show similarity with any other MT sequence. A dendrogram (Figure 9) of all the 60 MT sequences also illustrates the same pattern of results as obtained by these pairwise alignments. The two onnamide O-MTs in onnB and onnI genes and the N-MTs in pyochelin and anabaenopeptilide show clustering with C-methyltransferases. A stand alone O-MT in stigmatellin (stiK) is sequentially different from other O-methyltransferases, which was observed from the pairwise alignment results and is evident from the dendrogram. Thus it can be concluded from the above analysis that these 18 sequences can be used as templates to identify MT-domains in any given query sequence by pairwise alignment. These new MT templates were included in the current version of NRPS-PKS program for correct identification of various types of MT domains.

### Profile HMMs of C-, O- and N-methyltransferases

An alternative approach to detect MT domains in a new sequence is to query that sequence against a database of HMM profiles. Individual profiles were built for C-MT, O-MT and N-MTs using the curated sequences. These profiles were then used to make a HMM profile database for MT domains. The set of 61 C-MT, O-MT and N-MT sequences from experimentally characterized NRPS/PKS clusters were queried against this HMM database and the location of the MT-domain and their class was predicted in these sequences. Table 3 lists the score and E-value for alignment of 18 representative MT sequences with the HMM profiles of N-MT, C-MT and O-MT domains. As can be seen from Table 3, most C-MT sequences show statistically significant alignment with O-MT profiles and vice versa. On the other hand, only the N-MT sequences from pyochelin aligned with the N-MT as well as C-MT profiles unlike the other N-MT sequences which aligned with N-MT profile alone. This finding is consistent with results from pairwise sequence alignment discussed in the previous section.

**Table 3:** Scores and E-values for the alignment of 18 representative MT domains with the HMM profiles of N-MT, O-MT and C-MT.

	**NMT**		**OMT**		**CMT**	
	**Score**	**E-value**	**Score**	**E-value**	**Score**	**E-value**

actin01_NM_001	390.1	1.10E-117	-	-	-	-
anaba01_NM_001	252.8	2.40E-076	-	-	-	-
anaba01_OM_001	-	-	375.5	2.80E-113	-	-
barba01_OM_001	-	-	385.5	2.80E-116	-	-
bleom01_CM_001	-	-	**-54.3**	**4.70E-007**	385.5	2.80E-116
compa01_CM_001	-	-	-	-	332.7	2.10E-100
cyclo01_NM_001	449.7	1.30E-135	-	-	-	-
eryth01_OM_001	-	-	369.6	1.60E-111	-	-
leina01_CM_001	-	-	**-5.7**	**2.20E-010**	395.5	2.60E-119
melit01_OM_001	-	-	178.7	4.80E-054	-	-
nodul02_CM_001	-	-			270.3	1.30E-081
onnam01_OM_001	-	-	289.7	1.80E-087	**280.1**	**1.50E-084**
onnam04_OM_001	-	-	378.1	4.60E-114	-	-
peder01_OM_001	-	-	234.7	6.90E-071	-	-
pyoch01_NM_001	187.4	1.10E-056	-		**17.1**	**3.30E-013**
stigm03_OM_001	-	-	210.1	1.70E-063	-	-
thaxt02_NM_001	318.9	3.10E-096	-	-	-	-
yersi01_CM_002	-	-	-	-	321.4	5.20E-097

A '-'sign indicates that alignments resulted in scores with E-value higher than 1.0E-6. Several C-MTs align with O-MT profiles, while only N-MT of pyochelin synthase shows alignment with C-MT profile as well.

**Figure 10:**
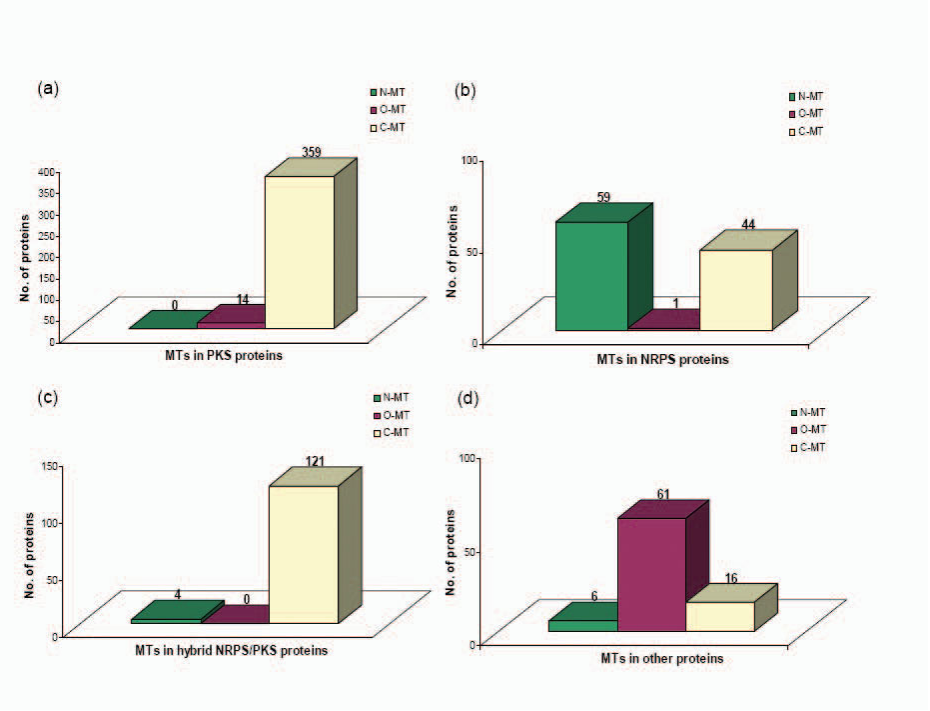
**Histograms showing the number of proteins in nr database having N-MT, O-MT and C-MT domains as identified by our HMM profile search.** (a) PKS proteins, (b) NRPS proteins, (c) hybrid NRPS/PKS proteins and (d) proteins other than NRPS/PKS proteins.

In order to test the predictive ability of our MT HMM profiles further, the recent version of the nr database of NCBI was also searched using these profiles to identify putative NRPS/PKS MT domains in various proteins. The sequences which matched with these profiles were grouped as C-MT, O-MT and N-MT containing sequences based on highest scoring profile match. The complete domain architecture of these MT containing proteins were also analyzed in details. The nr database search identified 4197 stand alone MT proteins and 684 multifunctional proteins containing MT domains. Out of the 4197 stand alone MT proteins, 3977 were O-MT proteins. In contrast to the very large number of stand alone O-MT proteins, there were only 155 stand alone C-MT and 55 stand alone N-MT proteins. Even though experimentally characterized NRPS/PKS biosynthetic pathways have a relatively larger number of stand alone O-MT proteins compared to stand alone C-MT or N-MT domains, it is not apparent whether all these stand alone O-MT proteins identified by our profile search would indeed be associated with secondary metabolite biosynthesis. Analysis of domain architectures in multifunctional proteins containing MT domains revealed several interesting results. These MT containing multifunctional proteins can be divided into four major groups. They are proteins containing MT domains along with core PKS domains, core NRPS domains, PKS as well NRPS domains and other catalytic domains. Figure 10 shows the number of proteins containing N-MT, O-MT and C-MT domains for each of the four categories. As can be seen from Figure 10a, 359 PKS proteins have C-MT domains, 14 PKS proteins have O-MT and none of them have N-MT domains. This result is consistent with the fact that polyketides have no sites for N-methylation and O-methylation in known PKS biosynthetic pathways is catalyzed by stand alone O-MTs. This indicates that our profiles are able to correctly classify the three different classes of secondary metabolite methyltransferases. Our nr database search also identified 59 NRPS proteins containing N-MT domains, 1 containing O-MT domains and 44 containing C-MT domains (Figure 10b). Since most nonribosomal peptide products are N-methylated, the presence of such a large number of C-MT domains as C-A-CMT-T modules was surprising. Similarly, the hybrid NRPS/PKS set also had 121 proteins containing C-MT domains as compared to only 4 proteins containing N-MT domains (Figure 10c). In fact 17 of these C-MT domains in hybrid NRPS/PKS proteins were present next to condensation domains of NRPS as C-CMT-PCP modules, while one would *a priori *expect N-MT domains in such modules instead of C-MT domains. In view of this finding of anomalously large number of NRPS modules containing C-MT domains in NRPS and hybrid NRPS/PKS proteins, we decided to analyze these proteins by pairwise alignment with 18 representative MT templates from known NRPS/PKS biosynthetic pathways. Interestingly the N-MT domain of pyochelin synthase was found to be closest match for 23 out of the 44 C-MT containing NRPS proteins identified by profile search. Most of these 23 NRPS proteins showed very high percentage identity with pyochelin synthase N-MT ranging from 27% to 69%. Thus it is very much likely that, these 23 NRPS proteins indeed contain pyochelin type N-MT domains which are different from other N-MT domains. They were annotated by profile approach as C-MTs, because N-MT domain of pyochelin synthase shows homology to C-MTs and has comparable scores with C-MT as well as N-MT profiles (Table 3). Similarly, a C-MT domain from yersiniabactin synthase was found to be the closest homolog of C-MTs found in NRPS modules of hybrid NRPS/PKS proteins and the sequence similarity was also very high. This MT domain in yersiniabactin synthase catalyzes C-methylation (Figure 2), but is present as C-CMT-PCP module similar to the domain organization found in C-MT containing hybrid NRPS/PKS proteins found by our profile search. This suggests that our profile search has genuinely identified yersiniabactin type novel C-MT domains embedded in NRPS modules. In case of 20 other NRPS proteins which showed matches with C-MT profiles, the C-MT domains from yersiniabactin, leinamycin and nodularin were found to be closest match. Table 4 shows the domain organization predicted for each of them by HMM profiles, and the percentage identity and similarity with closest matching MT domain in our 18 representative templates set. Even though the closest homolog approach also detects C-MT domains in these proteins in agreement with results from HMM approach, in view of the relatively low sequence identity with C-MTs from known NRPS/PKS biosynthetic pathways, it is not clear if these domains are likely to be genuine C-MT domains as in yersiniabactin synthase or a different class of N-MT domains which lack homology to known N-MT domains in our data set. Thus our analysis of nr database has revealed presence of many C-MT domains in NRPS modules as in yersiniabactin. It has also identified several pyochelin type N-MT domains which often show higher homology to C-MT profiles. None of these proteins are currently experimentally characterized. Experimental characterization of some of these MT domains would help in understanding how MT domains in NRPS/PKS family have evolved to acquire specificities for different substrates. However, a close examination of the chemical structures of pyochelin [37] and yersiniabactin [38] provides a rational for presence of N-MT domains with homology to C-MT sequences. As can be seen from Figure 2, in both these biosynthetic clusters MT domains transfer methyl groups to a five membered rings having very similar chemical structures. They only differ by the site of methylation. In view of the similarity in the structure of the acceptor substrate, pyochelin type N-MT domains show homology to C-MT proteins. It may be interesting to note that, in a recent study involving MTs from type II PKS pathways, Hertweck and coworkers [24] have observed that, these type II MTs cluster according to the site of methylation not necessarily as C-MTs, O-MTs and N-MTs. Based on this observation, they have proposed that for polyketide alkylation, regioselectivity is more dominant than the type of nucleophile (C-, O- or N-) that is being alkylated. Thus the C-MT domains found in NRPS modules by our profile search could indeed be due to such correlation between sequence of type I MTs and their regioselectivity. These observations have interesting implications of prediction of chemical structures of metabolites by genome mining.

**Table 4:** List of protein sequences from nr database which contain C-MT domains adjacent to the core NRPS domains

**Gi. no.**	**Domains**	**Identity (%)**	**Template**	**Organism name**
108809363	ACP-C-A-**CMT**-ACP-C	25	yersi01_CM_002	*Yersinia pestis Antiqua*
108809365	C-A-**CMT**	33	nodul02_CM_001	*Yersinia pestis Antiqua*
108813376	C-A-**CMT**	33	nodul02_CM_001	*Yersinia pestis *Nepal516
116215693	**CMT**-ACP-C-A-ACP-TE	47	yersi01_CM_002	*Vibrio cholerae *RC385
148271509	C-A-**CMT**-ACP	33	nodul02_CM_001	*Clavibacter michiganensis subsp.michiganensis*
153947007	C-A-**CMT**	33	nodul02_CM_001	*Yersinia pseudotuberculosis *IP 31758
153954130	ACP-C-A-**CMT**-ACP-C-ACP	29	nodul02_CM_001	*Clostridium kluyveri *DSM 555
16121089	C-A-**CMT**	33	nodul02_CM_001	*Yersinia pestis *CO92
16121091	ACP-C-A-**CMT**-ACP-C	25	yersi01_CM_002	*Yersinia pestis *CO92
17546530	C-A-**CMT**-ACP-TE	33	leina01_CM_001	*Ralstonia solanacearum *GMI1000
21225943	C-A-**CMT**-ACP	33	nodul02_CM_001	*Streptomyces coelicolor *A3(2)
22127285	ACP-C-A-**CMT**-ACP-C	25	yersi01_CM_002	*Yersinia pestis *KIM
26248281	ACP-C-**CMT**-ACP-TE	100	yersi01_CM_002	*Escherichia coli *CFT073
28869792	ACP-C-A-**CMT**-ACP-C-ACP	27	leina01_CM_001	*Pseudomonas syringae pv. tomato *str. DC3000
41409840	C-A-**CMT**	34	yersi01_CM_002	*M. avium subsp. paratuberculosis *K-10
45443170	C-A**-CMT**	33	nodul02_CM_001	*Yersinia pestis biovar Microtus *str. 91001
51597596	C-A-**CMT**	33	nodul02_CM_001	*Yersinia pseudotuberculosis *IP 32953
89102588	ACP-C-A-**CMT**-ACP-C	25	yersi01_CM_002	*Yersinia pestis biovar Orientalis *str. IP275
89895567	C-A-**CMT**	37	yersi01_CM_002	*Desulfitobacterium hafniense *Y51
90424526	C-A-**CMT**-ACP-TE	38	yersi01_CM_002	*Rhodopseudomonas palustris *BisB18

List of protein sequences from nr database which contain C-MT domains adjacent to the core NRPS domains as identified by our MT HMM profile search. Gene identifier number, domain organization and organism name is listed in columns 1, 2 and 5. Columns 4 and 3 list the closest match (as identified by pair BLAST) with the representative MT domains from experimentally characterized NRPS/PKS clusters and the corresponding percentage identities.

The analysis of MT containing proteins in nr database also revealed the co-occurrence of NRPS/PKS type MT domains with several other catalytic domains apart from core PKS and NRPS domains (Figure 10d). However, in most cases these proteins had O-MT domains and there were relatively few C-MT and N-MT domains. Table 5 shows domain organization for representative proteins from each category and also lists the number of such proteins identified by our HMM profile search. As can be seen, the O-MT domains are present along with a variety of catalytic domains which do not belong to core NRPS or PKS family. Interestingly 19 out of the 61 examples correspond to co-occurrence with glycosyltransferase domains. Since glycosyltransferases are a major class of tailoring enzymes involved in secondary metabolite biosynthesis, many of these proteins might indeed be associated with novel PKS/NRPS biosynthetic pathways [39,40]. Apart from glycosyltransferases, the other functional domains which occur with O-MT are oxidases, hydroxylases, tetratricopeptide repeat (TPR), phosphodiasterases, DNA binding helix-turn helix proteins etc. It would be necessary to further analyze each of these proteins in details to understand their biochemical function. In contrast to O-MTs, the N-MT and C-MT domains are present in multifunctional enzymes which contain other catalytic domains in addition to PKS and NRPS domains. Some of the interesting examples are NRPS/PKS proteins containing chalcone synthase or carnitine acyltransferase domains along with C-MT domains. Similarly, N-MT domains are present along with domains associated with streptococcal surface antigen, bacterial luciferase, phosphoenol pyruvate (PEP) utilizing enzyme and ketopantoate reductase (ApbA) etc. Thus these results give valuable clues about organisms which can potentially make secondary metabolites with a variety of structural modifications by diverse types of tailoring enzymes. Some of these proteins would be interesting targets for detailed experimental investigation.

**Table 5:** List of representative protein sequences from nr database which contain MT domains in combination with other functional domains

**Gi no.**	**Domains**	**No. of proteins**	**Organism name**
113477217	Glycos_transf_1-**OMT**-Glycos_transf_2	19	*Trichodesmium erythraeum *IMS101
110599935	TPR_1-TPR_2-TPR_1-TPR_2-TPR_2-TPR_1-TPR_2-**OMT**	18	*Geobacter *sp. FRC-32
154319269	KS-KS-AT**-CMT**-ER-DH-KR-Carn_acyltransf	7	*Botryotinia fuckeliana *B05.10
126178605	**OMT**-Dala_Dala_lig_C	4	*Methanoculleus marisnigri *JR1
110634799	Abhydrolase_1-**OMT**	3	*Mesorhizobium *sp. BNC1
31794901	Radical_SAM-**OMT**	3	*Mycobacterium bovis *AF2122/97
30681189	DUF248-**OMT**	2	*Arabidopsis thaliana*
62290714	HTH_3-**OMT**	2	*Brucella abortus biovar *1 str. 9–941
71013608	Amidohydro_3-**OMT**	2	*Ustilago maydis *521
107099798	FA_hydroxylase-**OMT**	2	*Pseudomonas aeruginosa *PACS2
147791135	Amino_oxidase-**OMT**	2	*Pseudomonas aeruginosa *PACS2
156064387	E1-E2_ATPase-**OMT**	2	*Sclerotinia sclerotiorum *1980
157382467	A-ACP-C-A-**NMT**-ACP-ACP-C-ApbA-ApbA_C	1	*Xylaria *sp. BCC 1067
157752876	**OMT**-Nol1_Nop2_Fmu	1	*Caenorhabditis briggsae*
153813751	AstE_AspA-**OMT**	1	*Ruminococcus obeum *ATCC 29174
71030506	**OMT**-Pox_MCEL	1	*Theileria parva *strain Muguga
66825109	KS-KS-AT-**CMT**-DH-KR-Chal_sti_synt_N-Chal_sti_synt_C	1	*Dictyostelium discoideum *AX4
145608084	HET-**OMT**	1	*Magnaporthe grisea *70-15
147858936	DUF642-**OMT**	1	*Vitis vinifera*
158520366	DUF1365-**OMT**	1	*Desulfococcus oleovorans *Hxd3
115402313	**OMT**-MIP	1	*Aspergillus terreus *NIH2624
110681402	C-A-**NMT**-ACP-C-A-ACP-TE-PEP-utilizers	1	*Chondromyces crocatus*
17546523	**OMT**-TE-ACPS	1	*Ralstonia solanacearum *GMI1000
51892502	Phosphodiest-**OMT**	1	*Symbiobacterium thermophilum *IAM 14863
116207616	MFS_1-KS-KS-AT-**CMT**-DH-ER-KR-DH	1	*Chaetomium globosum *CBS 148.51
41407518	C-A-Strep_SA_rep-ACP-C-A-**NMT**-ACP-C-A-Strep_SA_rep-ACP-C-A-Strep_SA_rep-ACP-C-A-Strep_SA_rep-ACP-TE	2	*Mycobacterium avium subsp*. *paratuberculosis *K-10
26541536	ACP-KR-DH-KS-KS-ACP-ACP-KS-KS-KR-DH-ACP**-CMT**-ACP-KS-KS-DH-KR-ACP-KS-KS-ACP-ACP-Beta_elim_lyase-Abhydrolase_1	1	*Streptomyces atroolivaceus*
108757966	A-ACP-C-A-**NMT**-ACP-C-A-ACP-C-A-ACP-C-A-ACP-C-A-ACP-KS-KS-AT-ACP-C-Bac_luciferase-C-A-ACP-C-A-ACP-C-A-ACP-C-A-ACP-C-A-ACP-TE	1	*Myxococcus xanthus *DK 1622

List of representative protein sequences from nr database which contain C-MT, N-MT or O-MT domains in combination with functional domains other than core PKS or NRPS domains. Gene identifier number, domain organization and organism names are listed in columns 1, 2 and 4 respectively, while column 3 lists total number of proteins having similar domain organization.

## Discussions

We have carried out a comprehensive analysis of the sequence and structural features of the MT domains present in multi functional proteins encoded by various experimentally characterized NRPS, PKS and hybrid NRPS/PKS gene clusters with known secondary metabolite products. Even though presence of methyltransferase domains in NRPS and PKS family of megasynthases have been inferred from methylation pattern of the chemical structure of the secondary metabolite product, earlier studies have not defined the correct domain boundaries. Threading analysis of the MT containing sequence stretches suggest 1VLM and 1VL5 as possible structural templates for NRPS/PKS MT domains. Based on the alignment with these structural templates, we identify the correct boundaries and predict that, the general length of MT domains will be in the range of 200–220 residues. Our threading analysis also reveals interesting homology between AT-MT and AT-DH linkers. Similarly, large sequence stretches C-terminus to the C-MT domains of PKS proteins are found to have homology with structural half of KR domains. These results not only explain the large variation in the length of the MT containing sequence stretch, they can also provide valuable clues for design of domain swapping experiments.

The curated sequences of these MT domains were further analyzed. From the MT sequences of correct length, a representative set of 18 MT domains covering the entire range of sequence divergence were chosen. A novel protocol for identification of MT domains by pairwise alignment and their classification as N-MT, C-MT and O-MT was developed using these 18 MTs as multiple templates. This MT domain identification protocol has been implemented in the current version of the program NRPS-PKS. Using this approach C-MT domains were annotated in the PKS proteins of *Dictyostelium discoideum *in a recent work [41].

The MT sequences with correct domain boundaries were also used to build profile HMMs for O-MT, C-MT and N-MT domains. Using these HMM profiles searches were carried out in the nr database of NCBI for identifying various other proteins containing C-MT, O-MT and N-MT domains. It is interesting to note that, apart from core PKS and NRPS domains, these secondary metabolite biosynthetic MT domains are also associated with other important catalytic domains like glycosyltransferase, oxidases, hydroxylases, phosphodiesterases and reductases. These proteins could be interesting targets for experimental characterization. Our analysis also surprisingly revealed the presence of a large number C-MT domains in NRPS modules adjacent to condensation (C) and adenylation (A) domains. These predicted C-MT domains can be classified into two groups. One group of C-MT domains showed high homology to N-MT domain of pyochelin synthase, which is different from typical N-MT domains present in NRPS modules. Unlike typical N-MT domains of NRPS proteins, this shows homology to C-MT domains but catalyzes transfer of methyl group to nitrogen. This group could indeed be pyochelin type N-MT domains, but are mis-classified as C-MT due to their homology with C-MT proteins and under representation of pyochelin type N-MT in our training data set. The closest homolog of the other group of C-MT domains present in NRPS modules is the C-MT domain of yersiniabactin synthase, which is present adjacent to a condensation domain of NRPS, but is indeed a C-MT. However, in view of the low homology between the C-MT from yersiniabactin and this second group of predicted C-MTs, it is difficult to predict whether they are yersiniabactin or pyochelin type MTs. A close examination of the chemical structures of the yersiniabactin and pyochelin provides an evolutionary basis for the presence of pyochelin type N-MT domains having homology with C-MT proteins. As can be seen from Figure 2, an identical five membered ring is the acceptor moiety for these two classes of MTs, while pyochelin N-MT methylates at the N position of the ring, the yersiniabactin C-MT methylates the adjacent C position. It may be interesting to note that such correlation between regioselectivity of the site of methylation and MT sequence have also been reported earlier by Hertweck and coworkers [24] for MTs in type II PKS biosynthetic pathways. Experimental characterization of proteins identified by our analysis would help in building more specific profiles for these novel MT domains.

## Conclusion

We have carried out a comprehensive bioinformatics analysis of methyltransferase (MT) domains present in PKS/NRPS clusters having known secondary metabolite products. Based on the site of methylation of these known secondary metabolites, the MT domains have been grouped as N-MT, C-MT and O-MT proteins and sequence/structural features have been analyzed in detail for each group. Based on the results of this analysis, we have developed a novel knowledge based computational approach for detecting MT domains present in PKS and NRPS megasynthases, delineating their correct boundaries and classifying them as N-MT, C-MT and O-MT using profile HMMs. Analysis of proteins in nr database of NCBI using these class specific profiles has revealed several interesting examples of MT domains with novel substrate specificities. Our analysis has also given interesting insight into the evolutionary basis of the novel substrate specificities of these MT proteins. These results have interesting implications for identification of novel secondary metabolites by genome mining and also rational design of novel natural products by biosynthetic engineering.

## Methods

For various PKS/NRPS clusters catalogued in NRPSDB [5], PKSDB [4] and ITERDB [5], the multi functional proteins containing MT domains were identified based on comparison of chemical structure of the metabolic products with the domain organization in the proteins of the corresponding biosynthetic clusters. The PKS/NRPS clusters in which the MT domains were found included actinomycin [32], metithiazol A [42], pyochelin [37], yersiniabactin [38], stigmatellin [43], anabaenopeptilide [44], enniatin [31], leinamycin [45], microcystin [46], jamaicamide A [47], complestatin [48], bleomycin [35], epothilone [49], myxothiazol [50], nodularin [51], pristinamycin [52], thaxtomin [53], tubulysin [54], onnamide [36], pederin [36], barbamide [55], cyclosporine [56], lovastatin [57], compactin [58], fumonisin [59], erythromycin [60] and equisetin [61]. The various MT containing multi functional proteins found in these PKS/NRPS clusters were analyzed by NRPS-PKS web server [5] as well as CDD [62] search. Both NRPS-PKS and CDD often failed to detect full length MT domains due to lack of homology over the complete length. The sequence stretch identified by these programs along with their flanking linkers was threaded on various structural folds in PDB using GenTHREADER [63] and PHYRE fold recognition servers [64]. In cases where chemical structure of metabolites indicated presence of MT domains but no MT domain was detected by these programs, all linker stretches having unusual length were analyzed by GenTHREADER. It is known that, in many NRPS clusters, N-MT domain is inserted between A-8 and A-9 conserved signature motifs of the adenylation domain. Therefore, if an adenylation domain produced two discrete regions of local alignments with a regular A domain, the unaligned stretch was analyzed by threading method for possible presence of MT domains. Apart from integrated MT domains present in multidomain proteins, 8 stand alone O-MT present in NRPS or PKS clusters were also analyzed by GenTHREADER.

In order to choose a minimal set of templates representing the entire range of sequence diversity, every sequence was compared with every other sequence using BLAST program [65]. Based on these alignments, sequences with similarity above 50% and alignment length greater than 90% were grouped together. A representative sequence was selected from each group and finally 18 sequences were selected as templates for identification of MT domains by pairwise alignment. These 18 templates included five C-MT, five N-MT and eight O-MT sequences.

The sequence to structure alignments obtained from GenTHREADER were sorted according to their threading score and the top-ranking hits were selected as possible structural templates. The boundaries of the predicted domains were obtained from the aligned region between the query and the crystal structure. The sequences were then classified into three groups as N-MT, C-MT and O-MTs by correlating with the structure of the metabolic product. The curated sequences of the three classes were taken and MSA was performed using ClustalW2 [66] and ESPript [67] software. MSA was used to study the similarity of the three classes of MT sequences and also find the pattern of conservation of the motifs among these sequences. A phylogenetic tree was obtained from multiple sequence alignment using iTOL [68]. Bootstrapping was performed 1000 times to obtain support values for each node. All significant bootstrapping values (more than 500) are shown in figure 7. A local version of HMMER 2.3.2 package was used to derive profile HMMs for each class of MT domains. These profiles were used to search the nr database (non-redundant database) of NCBI for identifying NRPS/PKS family of C-MT, N-MT and O-MT domains present in various proteins. The proteins showing matches with these HMM profiles were searched locally using Pfam – A database release 22.0 [69] at E-value cut off of 10^-6 ^for identifying other catalytic domains associated with C-MT, N-MT and O-MT.

## Authors' contributions

MZA and JS performed the computations, analyzed data and wrote the manuscript; RSG and DM designed research, analyzed data and wrote the manuscript. All the authors read and approved the final manuscript.

## Supplementary Material

Additional file 1 **MSWORD file containing supplementary Figure 1.** Click here for file

Additional file 2 **MSWORD file containing supplementary Table 1.** Click here for file
