# Supplementary material for: In silico analysis of methyltransferase domains involved in biosynthesis of secondary metabolites
Source: BMC Bioinformatics. 2008 Oct 25;9:454. doi: 10.1186/1471-2105-9-454 (PMC2613160; doi:10.1186/1471-2105-9-454)
Supplement: Additional file 1 — MSWORD file containing supplementary Figure 1. [file 1471-2105-9-454-S1.doc]

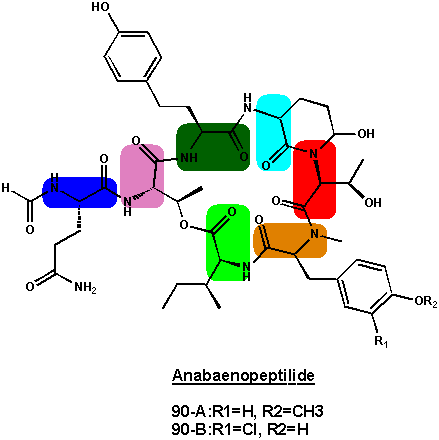

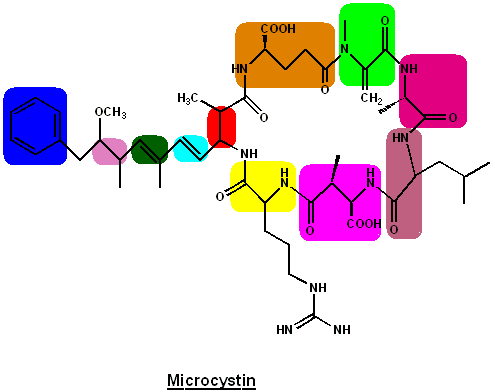

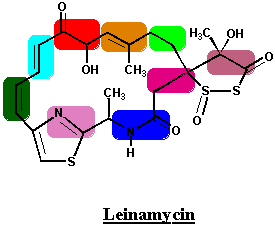

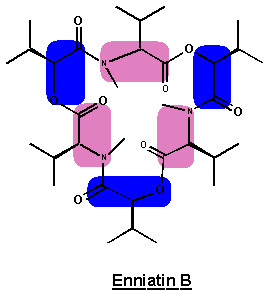

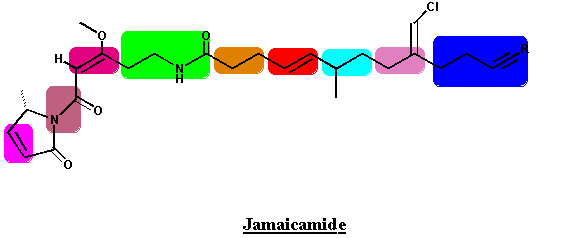

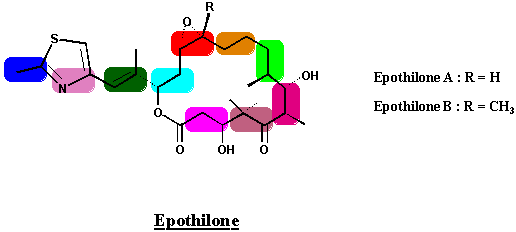

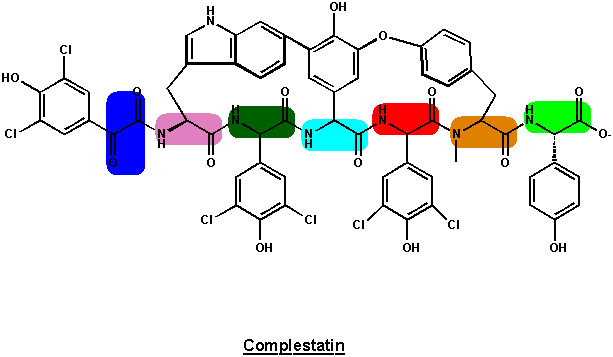

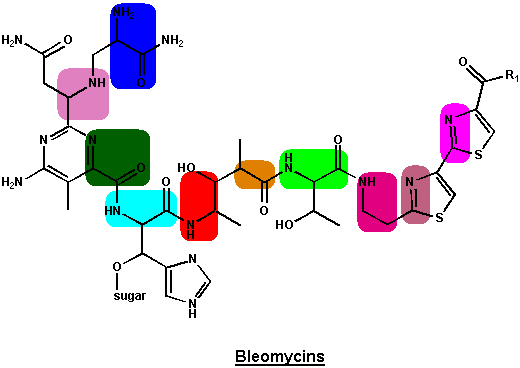

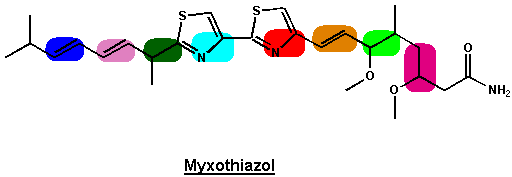

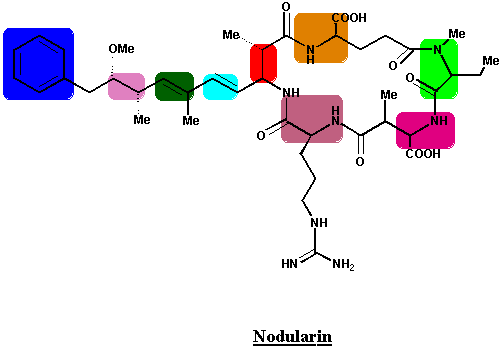

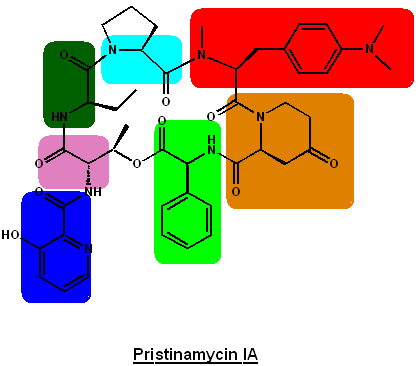

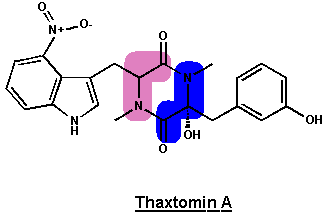

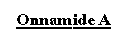

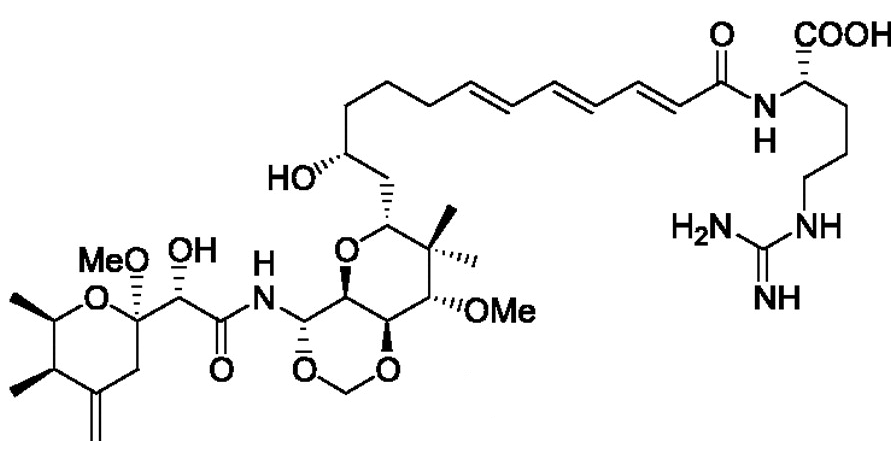

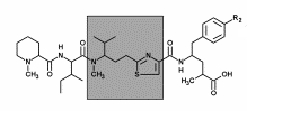

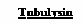

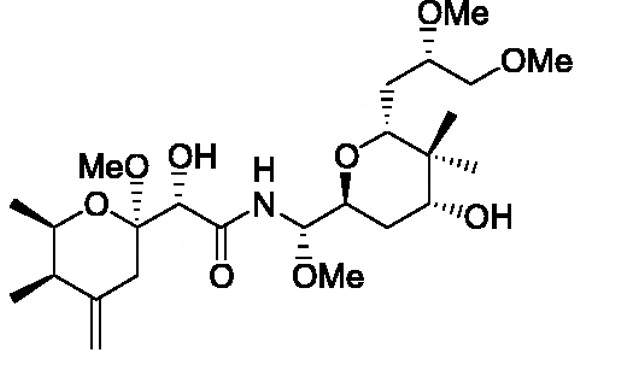

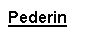

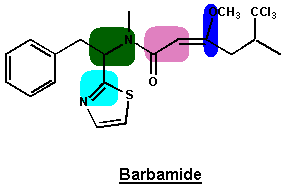

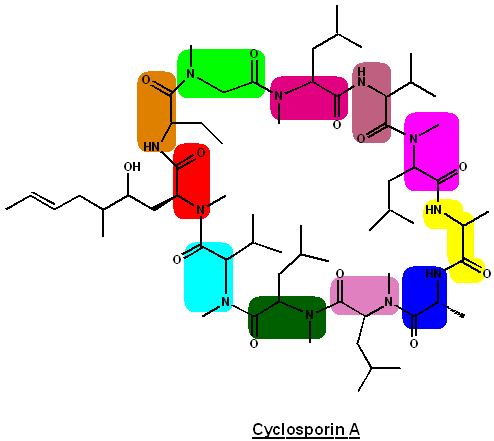

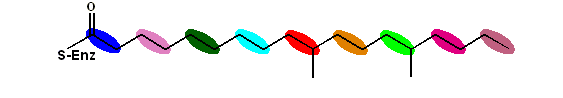

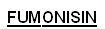

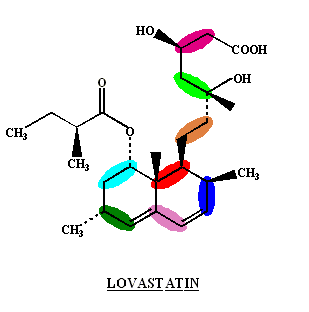

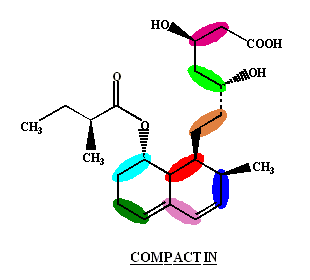

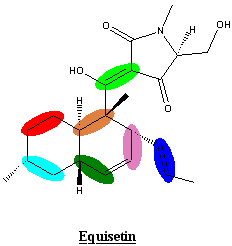

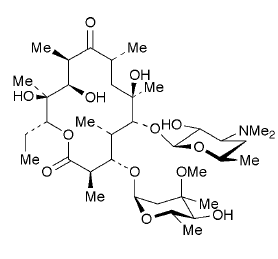

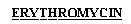


**Figure S1 :** Chemical structures of 22 secondary metabolites containing methyl groups (highlighted by arrow sign) added by C-MT, N-MT and O-MT enzymatic domains. The secondary metabolites biosynthesized by PKS/NRPS clusters are anabaenopeptilide, enniatin, leinamycin, microcystin, jamaicamide A, complestatin, bleomycin, epothilone, myxothiazol, nodularin, pristinamycin, thaxtomin, tubulysin, onnamide, pederin, barbamide, cyclosporine, lovastatin, compactin, fumonisin, erythromycin and equisetin.
