# Supplementary material for: In silico analysis of methyltransferase domains involved in biosynthesis of secondary metabolites
Source: BMC Bioinformatics. 2008 Oct 25;9:454. doi: 10.1186/1471-2105-9-454 (PMC2613160; doi:10.1186/1471-2105-9-454)
Supplement: Additional file 2 — MSWORD file containing supplementary Table 1. [file 1471-2105-9-454-S2.doc]

**Supplementary Table 1:** The length of 61 N-MT, C-MT and O-MT containing sequences and other domains flanking the MT domain.

| **Methyltransferases** | **Length** | **Domain organization** |
| --- | --- | --- |
| **actin01_NM_001** | **422** | **C-A-MT-T** |
| **actin01_NM_002** | **402** | **C-A-MT-T** |
| **anaba01_NM_001** | **367** | **C-A-MT-T** |
| **anaba01_NM_002** | **406** | **C-A-MT-T** |
| **barba01_NM_001** | **401** | **C-A-MT-T** |
| **compl01_NM_001** | **420** | **C-A-MT-T** |
| **cyclo01_NM_001** | **431** | **C-A-MT-T** |
| **cyclo01_NM_002** | **449** | **C-A-MT-T** |
| **cyclo01_NM_003** | **406** | **C-A-MT-T** |
| **cyclo01_NM_004** | **464** | **C-A-MT-T** |
| **cyclo01_NM_005** | **444** | **C-A-MT-T** |
| **cyclo01_NM_006** | **458** | **C-A-MT-T** |
| **cyclo01_NM_007** | **405** | **C-A-MT-T** |
| **ennia01_NM_001** | **454** | **C-A-MT-T** |
| **micro01_NM_001** | **416** | **C-A-MT-T** |
| **nodul01_NM_001** | **406** | **C-A-MT-T** |
| **prist01_NM_001** | **411** | **C-A-MT-T** |
| **pyoch01_NM_001** | **390** | **C-A-MT-T** |
| **thaxt01_NM_001** | **428** | **C-A-MT-T** |
| **thaxt02_NM_001** | **362** | **C-A-MT-T** |
| **tubul01_NM_001** | **399** | **C-A-MT-T** |
| **tubul02_NM_001** | **442** | **C-A-MT-T** |
| **bleom01_CM_001** | **640** | **KS-AT-MT-KR** |
| **equis01_CM_001** | **700** | **KS-AT-MT-KR** |
| **micro03_CM_001** | **723** | **KS-AT-MT-KR** |
| **nodul01_CM_001** | **700** | **KS-AT-MT-KR** |
| **tubul01_CM_001** | **789** | **KS-AT-MT-KR** |
| **yersi01_CM_001** | **728** | **KS-AT-MT-KR** |
| **yersi01_CM_002** | **479** | **C-MT-T-TE** |
| **peder01_CM_001** | **515** | **KS-AT-MT-ACP** |
| **epoth01_CM_001** | **803** | **KS-AT-DH-MT-KR** |
| **jamai01_CM_001** | **748** | **KS-AT-DH-MT-ER-KR** |
| **nodul02_CM_001** | **720** | **KS-AT-DH-MT-KR** |
| **micro01_CM_001** | **791** | **KS-AT-DH-MT-KR** |
| **compa01_CM_001** | **490** | **KS-AT-DH-MT-KR** |
| **compa02_CM_001** | **713** | **KS-AT-DH-MT-ER-KR** |
| **fumon01_CM_001** | **711** | **KS-AT-DH-MT-ER-KR** |
| **lovas01_CM_001** | **571** | **KS-AT-DH-MT-ER-KR** |
| **lovas02_CM_001** | **706** | **KS-AT-DH-MT-ER-KR** |
| **leina01_CM_001** | **432** | **KS-DH-KR-ACP-MT-ACP** |
| **micro02_CM_001** | **506** | **KS-AT-ACP-MT-AMT-C-A-T-C** |
| **nodul03_CM_001** | **442** | **KS-AT-ACP-MT-AMT-C-A-T-C** |
| **melit01_OM_001** | **609** | **KS-AT-MT-KR** |
| **myxot01_OM_001** | **644** | **KS-AT-MT-KR** |
| **stigm01_OM_001** | **624** | **KS-AT-MT-KR** |
| **stigm02_OM_001** | **659** | **KS-AT-MT-KR** |
| **anaba01_OM_001** | **263** | **Stand alone MT** |
| **jamai01_OM_001** | **488** | **Stand alone MT** |
| **nodul01_OM_001** | **309** | **Stand alone MT** |
| **onnam02_OM_001** | **317** | **Stand alone MT** |
| **onnam03_OM_001** | **321** | **Stand alone MT** |
| **onnam04_OM_001** | **268** | **Stand alone MT** |
| **peder01_OM_001** | **312** | **Stand alone MT** |
| **peder02_OM_001** | **273** | **Stand alone MT** |
| **stigm03_OM_001** | **256** | **Stand alone MT** |
| **eryth01_OM_001** | **306** | **Stand alone MT** |
| **barba01_OM_001** | **422** | **KS-AT-MT-ACP** |
| **melit02_OM_001** | **407** | **KS-AT-MT-ACP** |
| **onnam01_OM_001** | **484** | **KS-AT-MT-ACP** |
| **onnam05_OM_001** | **491** | **KS-AT-MT-ACP** |
| **myxot02_OM_001** | **507** | **KS-AT-MT-ACP** |
